# Universal WASH coverage; what it takes for fragile states. Case of Jariban district in Somalia

**DOI:** 10.1371/journal.pone.0247417

**Published:** 2021-02-25

**Authors:** Wonder Mafuta, Jethro Zuwarimwe, Marizvikuru Mwale

**Affiliations:** Institute of Rural Development, University of Venda, Thohoyandou, South Africa; Universidad Nacional Autonoma de Nicaragua Leon, NICARAGUA

## Abstract

The paper assessed access to WASH service in the Jariban district of Somalia. One hundred and sixty-seven households were sampled to administer a questionnaire. Central tendency and logistical regression were used to analyse the data in SPSS 26. The findings show that access to safe drinking water sources is 57.5%. Of the 42.5% of respondents who did not access safe drinking water source, only 10.8% confirmed that they treat drinking water at the point of use. The main reason for household water treatment was the positive mindset (.272) of the household head towards water treatment. The majority (80.2%) of the respondents access approximately 13 litres per person per day. Woman-headed households were more likely to treat water before drinking than male-headed households. Only 26.9% of the respondents accessed basic sanitation. Of the respondents, 55.7% did not share latrines, while 44.3% share resulting in open defecation. WASH access in the study area remains low, resulting in health-related risks, including diarrhoeal disease. The limitation is that the paper only focused on access to WASH facilities in fragile contexts. A cross-sectional analysis of biological, physical and chemical properties of water at the source and point of use is recommended for further research.

## 1.0 Introduction

Water, sanitation and hygiene are fundamental human rights. The benefits of access to water are fully realised when there is also access to adequate sanitation and good hygiene practices. Access to water, sanitation, and hygiene are interlinked and essential for achieving other development goals, including eliminating poverty and hunger and promoting equality. The efforts to reach universal coverage and ’leave no one behind’ are now under threat, particularly in fragile areas endowed by conflicts and humanitarian situations. Since 2005, there have been many disasters across the globe either of human and natural causes that have resulted in substantial damage to the already compromised WASH services and related WASH infrastructure [1]. These catastrophes have resulted in communities being displaced internally and across international borders, which further compromised WASH services’ access and sustainability. The priority of WASH services providers during emergencies is mostly on lifesaving with limited emphasis on long term sustainable interventions. While the MDG number 7 and SDG number 6 imperatives should be commended for prioritising access to WASH services, the sustainability of WASH services over time remains a challenge, particularly in fragile communities [2]. The paper assessed access to WASH service in a fragile war-torn country in line with the Sustainable Development Goal (SDG) number six and sphere guidelines. The types of water supply and sanitation technologies were investigated, including behaviours underpinning WASH infrastructure development, access and use.

### 1.1 Literature review

#### 1.1.1 Access to WASH facilities

The global SDG indicator ’proportion of the population using safely managed drinking water services’ is defined as an improved drinking water source: located on-premises available when needed, and compliant with faecal and priority chemical standards [3]. Improved WASH access can be achieved holistically by examining factors that change spatially and temporal. The transition to universal access is influenced by; socio-economic and political context, the structure of the economy, distribution of the population, ability to administer and level of solidarity within the country, and the government’s stewardship role [4]. Climate change and civil disruptions also affect the access to WaSH services and should be considering when building operational, legislative and institutional frameworks [3]. Studies have shown that access level across WASH components varies. For example, in health facilities, water supply is 51%, sanitation 23%, and hygiene data was not available and adequately monitored [5]. WASH assessment results are not composite in low and middle-income countries (LMICs) where only 2% of the health care facilities provided water, sanitation, hygiene and waste management services [6]. WASH access in fragile areas remains low, resulting in health-related risks, including diarrhoeal disease [7]. Diarrhoeal diseases mostly affect children under five resulting in high mortality rates worldwide [7]. Poor access also contributes to childhood undernutrition [8], maternal mortality [9] neglected tropical diseases [10], [11] and respiratory infections [12].[13]

#### 1.1.2 Combined WASH interventions

In SSA the effects of failing to combine WASH access is reflected through statistics; improved sanitation is 41.5%, 15.4% have improved water and sanitation without improved hygiene, and 4.4% of the SSA population have access to combined SDG access [14]. Attempts have been to assess WASH in a holistic sense in India [15]. A recent study has shown that combined WASH interventions, such as a water intervention with either hygiene education and improved sanitation improve access [12]. It is important to construct handwashing stations where there is a water supply system or existing latrines. Several authors posit the importance of promoting handwashing with soap to reduce diarrhoea [16] [17]. These studies confirm that hygiene facilities are a barrier to achieving combined SDG access.

#### 1.1.3 Responsive regulatory and institutional frameworks

Regulatory and institutional frameworks should accommodate and protect all stakeholder’s interests to ensure that WASH access is sustainable [18]. [19] posits that well crafted and policed frameworks ensure access to safe, reliable and sustainable drinking water. The specific policies should reflect the ethos of the international community, national governments, and the community. WASH governance at both macro and micro-level should be well articulated to improve WASH access. Micro-level governance refers to,

"…the range of political, social, economic and administrative systems that are in place to develop and manage water resources and the delivery of water services, at different levels of society."

The definition implies that WASH governance should have the transformative capacity to manage and cope with society changes [20]. Robust implementation of water governance eliminates inequality, ill health, poverty and underdevelopment [18].

## 2.0 Methods

Jariban district is located within the northeastern Puntland State of Somalia in Nugal region. The University of Venda approved the ethical clearance for the research under project number SARDF/20/IRD/02/0704. The Ministry of Planning, Economic Development and International Cooperation in the Puntland State of Somalia issued the research approval letter (MoPEDIC 067/02/20). The village leaders provided verbal consent for village participants. The data was collected between February and March 2020. A household survey was conducted through a structured questionnaire. The structured questionnaire had both ordinal and nominal questions. Access to different water supply and sanitation technologies was investigated using the questionnaire, including behaviours underpinning WASH infrastructure development, access and use.

Jariban district was purposively selected as it is ranked highest on the WASH severity index among the districts in Puntland state [21]. The WASH need in Somalia is defined by the WASH cluster severity index comprising five levels; (1). minimal (2). stress, (3). severe (4). extreme (5) catastrophic. Jariban district is in severity index five and ranked seventh of the 99 districts in federal Somalia [21].

A multi-stage sampling approach was adopted. The Krejci and Morgan (1970) sampling formula was used to calculate the sample size [22].

n=N/1+Ne2

Where;

n = Sample Size

N = Population size (number of households from)

e = Confidence interval (0.05).

Villages were randomly selected using the random number generator based on the village list. Households were also randomly selected from the sampled villages using the random number generator based on the household lists. The proportionate to population size (PPS) sampling technique was used to ensure villages with higher household numbers had a bigger sample compared to those with fewer households. Respondent identification was then conducted using a simple random sampling technique.

One hundred and sixty-seven self-administered household questionnaires were administered in the 19 villages. The questionnaires had a Likert scale indicating the infrastructure types, distance and time away from household and functionality. IBM Statistical Package for the Social Sciences version 26 was used for quantitative statistical analysis. Normality tests were conducted using Kurtosis and Skewness, and data was assumed normal since skewness was within range of +/-2 and kurtosis +/-3. The study’s validity and reliability were tested using the Cronbach Alpha, and variables determining water treatment behaviour were recorded at 0.840. The data were assumed as fit for logistic regression. Logistic regression (binary outcome) was applied to describe data and explain the relationship between dependent binary variables to independent variables. The dependent variable was household water treatment. The independent variables were the amount of water a household access each day, the cost of treating water and the perceived importance of water treatment. The three independent variables were chosen because they influence water treatment at Point of Use (PoU) or household level. The household-heads would only respond to issues that they had control over, such as access to water and affordability of treatment options. Chi- test was used to analyse the association of the role of women in water treatment.

## 3.0 Results

**Fig 1:**
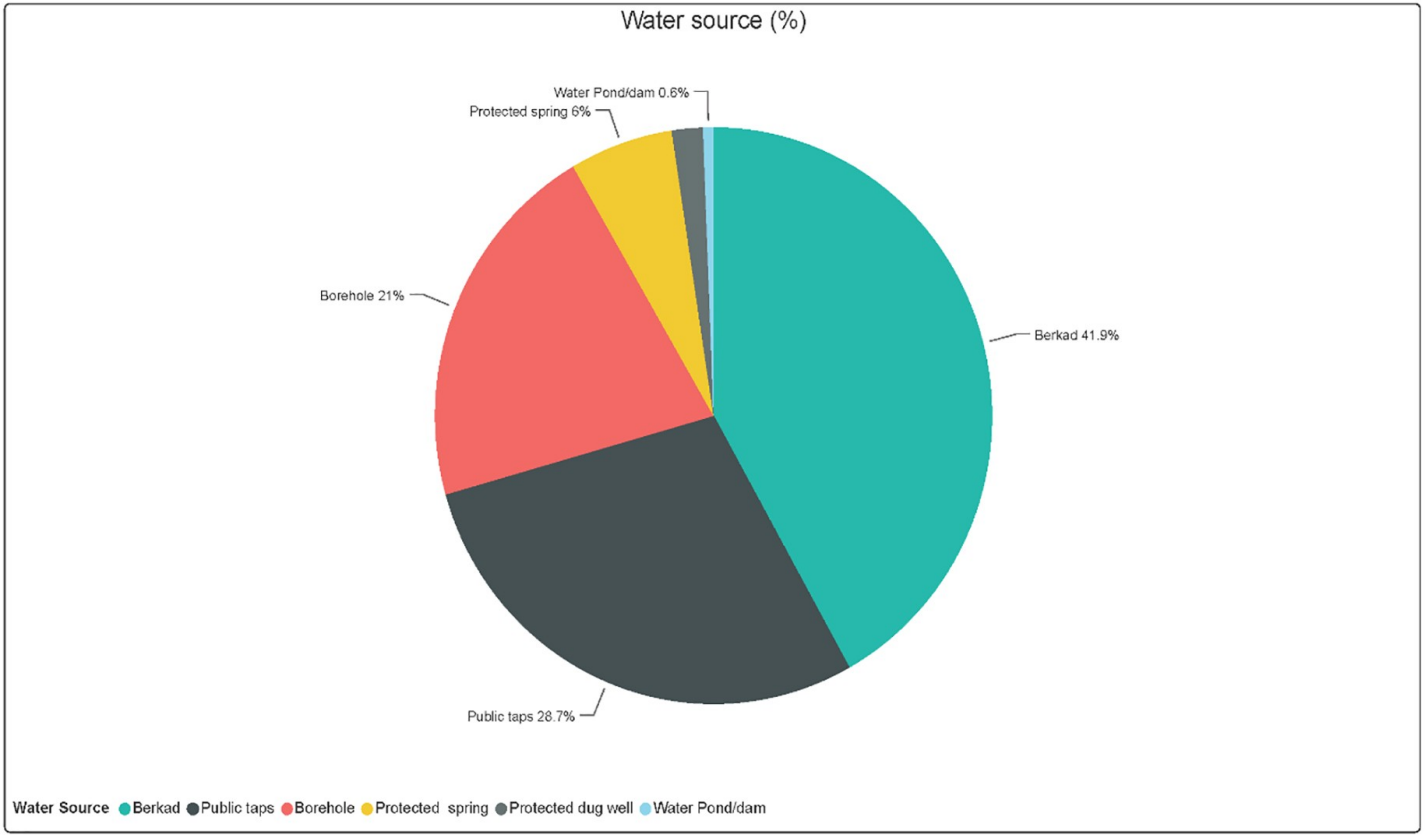
Types of water sources.

Concerning the source of water used by the respondents, the study revealed that the primary sources of water were the following; borehole (21%), public taps (28.7%), protected springs (6%), water ponds (0.6%), berkads (41.9%) and others 1.8%. The primary sources are shown in Fig 1.

**Fig 2:**
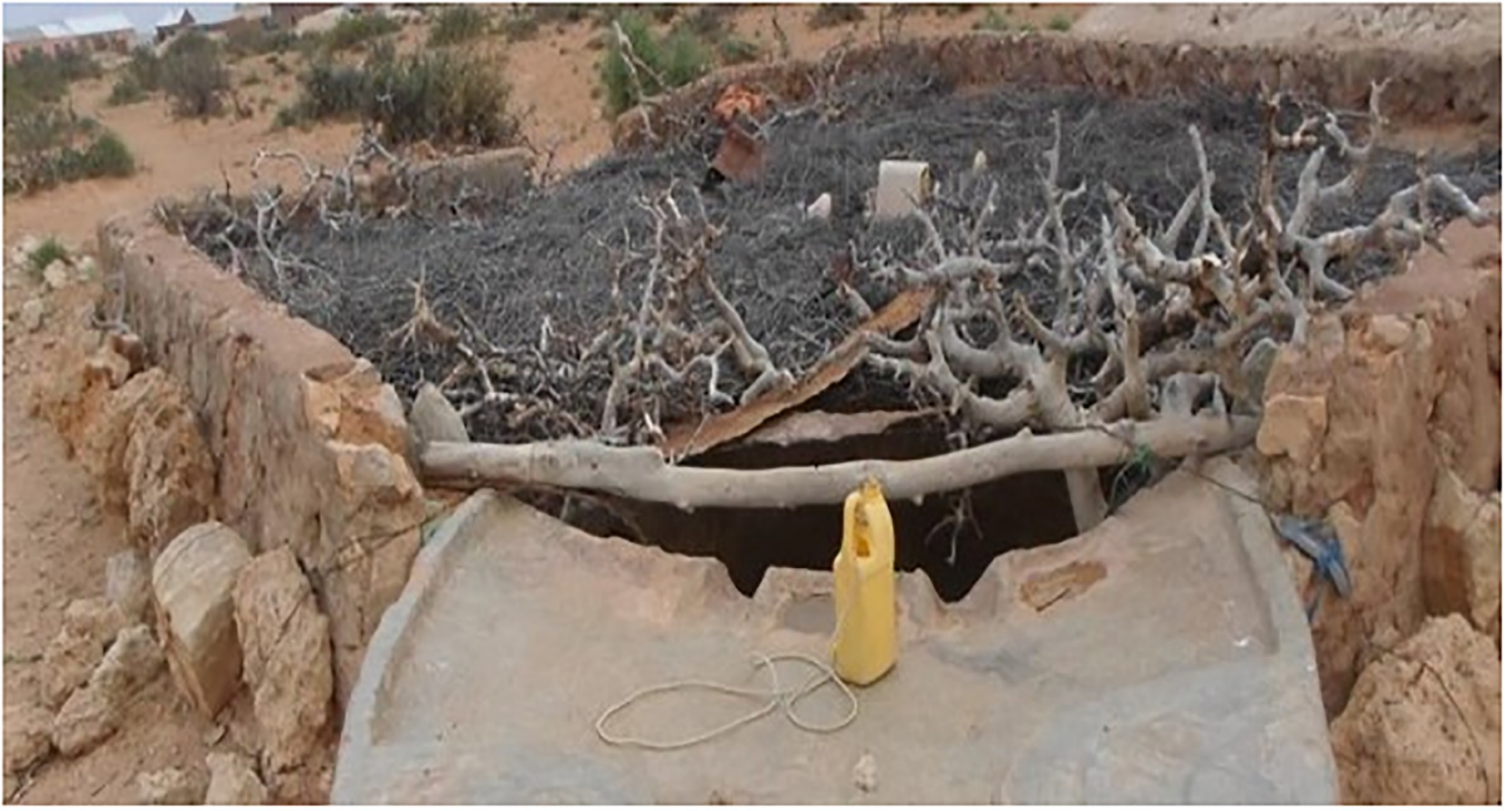
Picture of a berkad.

The main water source was berkads (41.9%). A berkad, as shown in Fig 2, is a water reservoir used in arid areas to collect water during the rainy season for use in the dry season. The water in berkads is harvested from runoff. When households buy water from water truckers, it is also stored in these berkads. Berkads are not protected or covered, thereby exposed to contamination.

Only 10.8% of the respondents confirmed that they treat drinking water at the point of use (POU). Out of the 10.8% who treated water before drinking; 9.6% boiled while 1.2% used chlorine products. The 10.8% also had different responses on how often they treated drinking water. The responses were rarely (44.1%), sometimes (44.1%) and always (11.8%). The majority of respondents (45.5%) acknowledged the importance of treating water, 41.3% (not important), 12.6% (very important) and 0.6% (not sure). The 89,2% respondents who did not treat water before drinking noted several reasons. The reasons were; chlorine materials were not accessible (12.5%), boiling was time-wasting (25%), and that there was no need to treat the water as it is clear (62.5%). The community’s responses established that members had no idea of water quality parameters (biological, physical, and chemical). Concerning the limited knowledge of water quality parameters, the study observed a high probability that water from boreholes could get contaminated when transported over long distances to the intended users as hygiene practices along the transportation route increased chances of contamination through handling.

The majority of the respondents (80.2%) noted that a household accessed 80 litres per day on average. Household size is, on average, six people in Somalia [23]. This means approximately 13 litres per person per day than the sphere indicator, which recommends 15 litres per person per day in humanitarian settings. As communities in Jariban are pastoralists, the water they get in a day is usually shared with domesticated animals like goats. Sharing the available water with livestock further dwindles the among of water each person access in a day.

Water sources were located less than 500 meters away for 97.6% of the respondent households. The Sphere indicator standards recommend that a water point should be less than 500 meters away from the homestead. Only 2.4% get water from a water source that is between 500 meters and 1000m away from the homestead. The sphere indicator recommends that the time needed for travelling and queueing time at a water point should be less than 30 minutes. The majority (95.8%) indicated that it takes them less than 30 minutes while only 4.2% queue for more than 30 minutes. The short distance to collect and queue for water was because the trucks distribute water close to the households.

**Fig 3:**
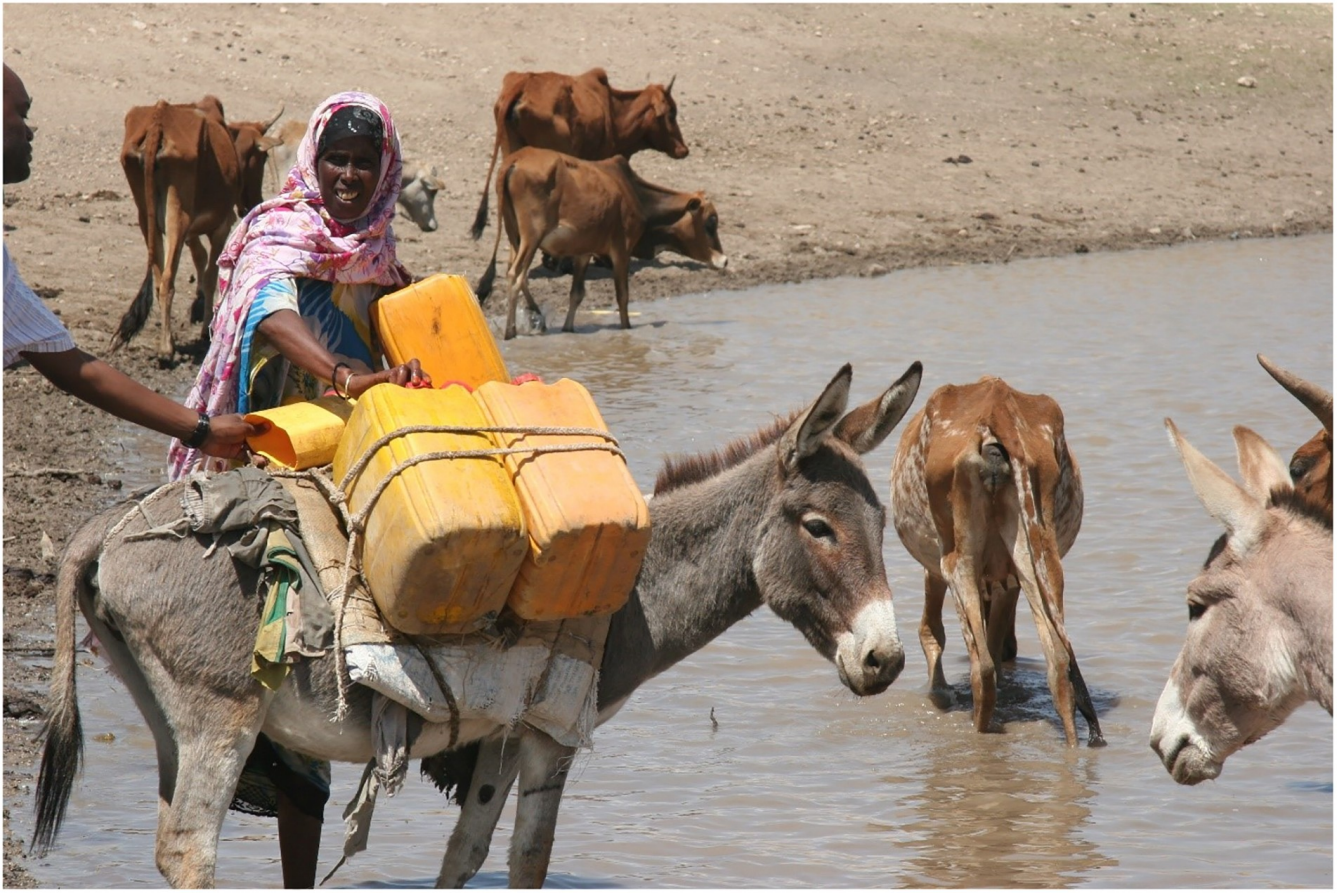
Picture of a pond where livestock and humans access water.

Of all the respondents, 79% indicated that water was always available and needed throughout the year. This could have been because water truckers were consistently bringing the water and that possibly the households were paying for the services on time. Over the year, limited access to water by 21% of the respondents implies that households access water from unprotected open ponds. The open ponds received water after short intermittent rains, and the water is finished in less than a month. Most of the water captured in these ponds is lost through filtration (given the loose sandy soils) and evaporation (due to high temperatures). The massive flock of camels, sheep, and goats drink from the same ponds resulting in the supply being outpaced by demand. The water is of poor quality, exposing populations to WASH-related diseases. Fig 3 shows a typical pond where households access water.

Of the 42.5% of respondents who did not access safe drinking water sources, only 10.8% confirmed that they treat drinking water at the point of use. Out of the 10.8% that treated water before drinking; 9.6% boiled while 1.2% used chlorine products. The 10.8% also had different responses on how often they treated drinking water. The responses were rarely (44.1%), sometimes (44.1%) and always (11.8%). The respondents who did not treat water before drinking cited several reasons: chlorine materials were not accessible, boiling was time-wasting, and there was no need to treat the water if it was clear. The responses that were given for not treating water show that the respondents had little knowledge about water treatment.

Logistic regression in SPSS version 26 was conducted to establish the relationship of factors why 89.2% of the respondents were not treating water. Three different variables were analysed, which are; water accessed per day (WT805), the cost of treating water (WT813), and the perceived importance of water treatment (WT814). Binary outcomes were calculated to identify each factor’s scale and magnitude, as shown in Table 1.

**Table 1:** Factors influencing household water treatment.

Variable	β	Sig.	Exp (β)
Household litres per day (WT805)	-.001	.835	.999
Cost of treating water (WT813)	-.021	.613	.979
Perceived importance of water treatment (WT814)	.272	.466	1.312
Constant	1.786	.018	5.966

^a^. Variable(s) entered on step 1: WT805, WT813, WT814

The amount of water a household accesses a day was less likely (-.001) to trigger people to treat water, the main reason being that households always accessed little water each day. The price of chlorine treatment packets was less likely (-.021) to improve household water treatment behaviour as the few chlorine tablets were not available in the local shops. However, water treatment at the household level was more likely to happen when the household head had a positive mindset (.272) towards water treatment. A Chi-Square test was conducted to determine whether water treatment is dependent on the gender of the household head. Results (16.352^α^) showed that water treatment depends on the gender of the household head. The relation was significant (ρ = .000). Where the household head was female, the likelihood of water treatment at the point of use was high.

**Fig 4:**
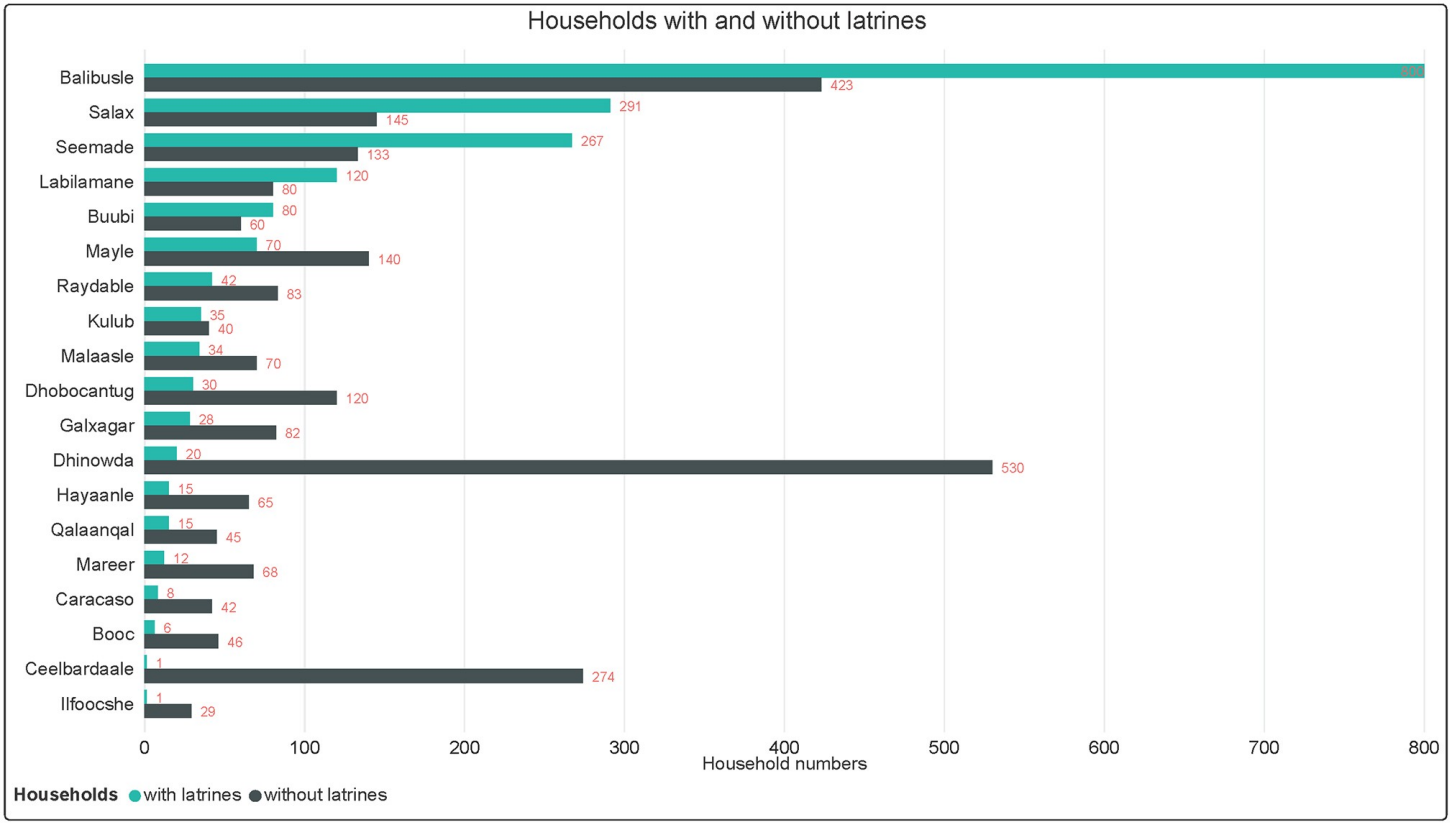
Number of households with and without latrines in 2020.

Latrines were counted in the target 19 villages, and the numbers are shown in Fig 4. In large villages (Balibusle, Salax, Seemade, Labilamane, and Buubi), households with latrines were more than those without latrines. However, in smaller villages, households without toilets were more than those with toilets. In larger communities, the settlement patterns were clustered and congested, making it difficult to defecate in the open.

**Fig 5:**
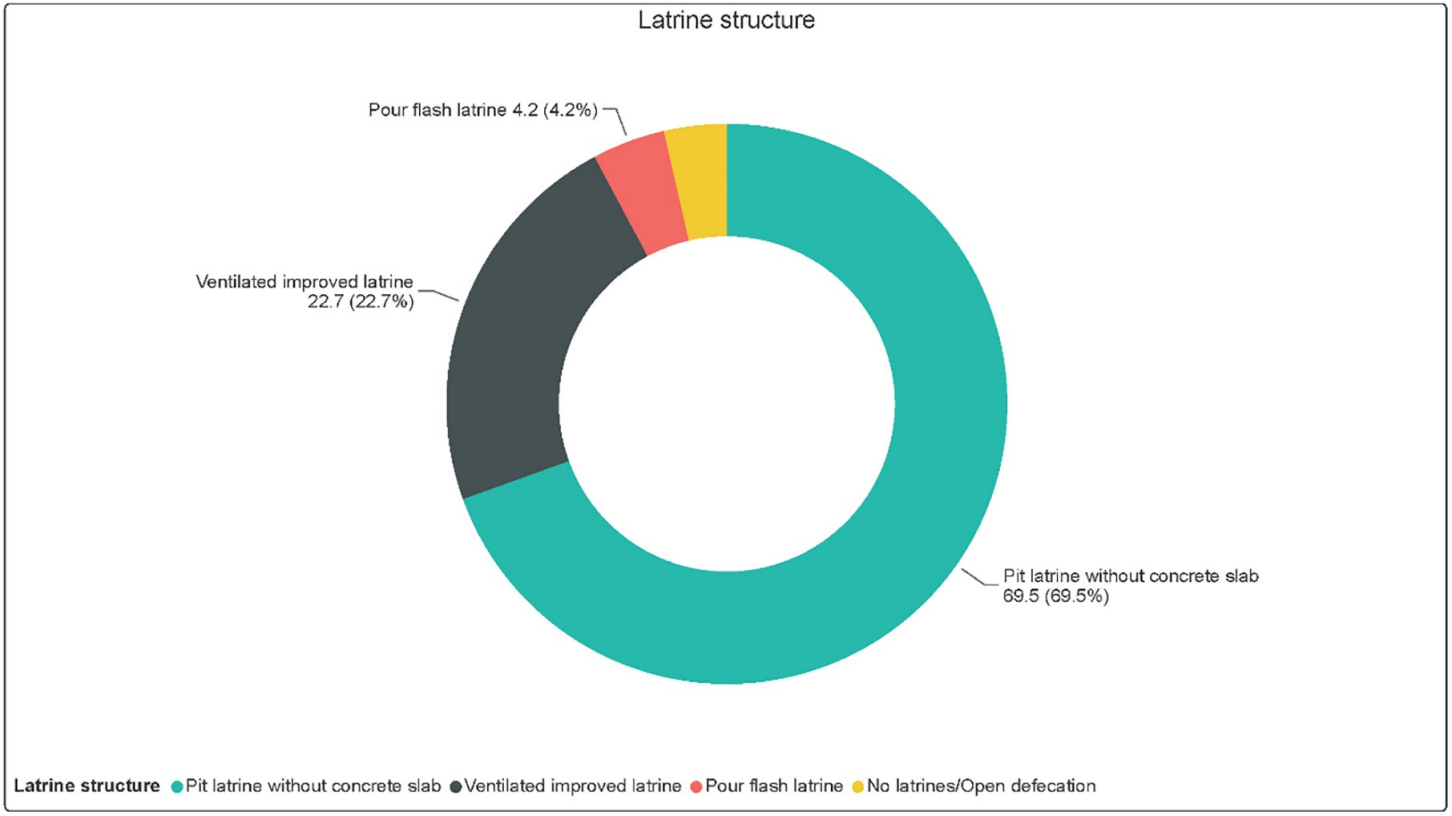
Types of latrines.

Pits latrines without concrete slabs were the majority being used (69.5%). The other types of latrines were the ventilated improved (22.7%), pour flash (4.2%), and open defecation (3,6%). Fig 5 shows the types of latrines in the study area. A pit latrine in the context of Jariban is a toilet with just a small excavated hole and a superstructure made of mud, plastic, and wood. Pour flush latrines had offset pit, which could be poured water after defecation. Concerning the SDGs target for sanitation, most of the latrines in Jariban do not meet the minimum requirements for improved, basic, or safely managed latrines.

Of the respondents, 55.7% did not share latrines, while 44.3% share. The sharing of latrines was not acceptable in the case of Jariban due to limited water supplies. In the 19 villages that were studied, only 1875 improved latrines were observed. Latrine user ratio of 1:62 was by far too high in relation to sphere guidelines [24] that recommends 20 people to share a communal latrine in emergencies. Of the respondents, 42.5% had no handwashing facility in place, 31,7% had a handwashing station with water only, 10.8% handwashing with water and ash, 8.4% handwashing station with no water and soap, 3.6% handwashing with soap and water while for 3% permission to observe was withheld.

## 4.0 Discussion

Due to limited boreholes in the district, some people (1.8%) resorted to buying bottled water or fetching from unprotected open sources (0.6%), thereby exposing them to Acute Watery Diarrhea (AWD). Data from the literature shows that unsafe water consumption impairs human health through illness caused by cholera, diarrhoea and typhoid [18] [19] [25].

The village settlement patterns in the study area were clustered. Other studies noted that in high and medium populated, as is the case of studied villages, the quality of water on major parameters is low [26]. The finding resonates with an earlier study that realised the need to balance access and quality issues in WASH service delivery, particularly in the developing world [27]. An earlier finding also confirmed females’ importance in dealing with contamination challenges and deep-rooted challenges such as arsenic [28]. In the context of Somalia, where the government and legislative system are weak, the water quality system is adversely affected. Low water treatment is confirmed by an earlier study that noted that achieving universal access remains a challenge, and efforts should be directed at attaining good health status and addressing equity, livelihoods, and education [2].

Low water access confirms earlier findings that developing countries in Sub-Saharan Africa (SSA) have more populations without access to clean water than other regions globally [27]. Kenya and Tanzania were reported to have 59% and 50% access to water, respectively, in earlier studies [29]. In Somalia and the rest of SSA, access to clean water is hampered by natural hazards, weak WASH governance/institutions, and the uncertain political will to address scarcity in the long run [27].

A study conducted in northern Ghana [30] also affirmed that open defecation was reduced where latrine construction approached completion. In other words, if a latrine was semi-covered, without lockable doors as was the case in Jariban, women were not comfortable using them, particularly at night. While sharing of latrines would reduce infectious diseases, emphasis should not be just on latrine ownership but rather proper and effective use of the latrines [31] [32]. Latrine type also determined the sharing of latrines, as noted in a recent study conducted in Ethiopia. The study revealed that latrines were used more if they had a superstructure, lockable door, and kept clean [33]. It has been noted that sanitation access has been regarded as the key measure in reducing infectious diseases [20]. Earlier studies note that latrines’ construction begins with behaviour change, and communities are triggered to build latrines [34]. When communities stay longer together, they handle land rights issues, resolve differences amongst social groups, and become economically empowered to the point that they invest in latrines. It should be noted that Muslims wash hands with soap before and after they pray (ablution). As much as they may be no handwashing stations outside the latrines, soap and water are used to wash the body before prayers critically. Handwashing is essential in reducing the transmission of bacteria and viruses between individuals [29].

## 5.0 Conclusion and recommendations

The respondents in the study area access little water per person per day as compared to sphere guideline. The distance travelled to the water source was within the range of less than 500 meters as specified by the SDGs. Access to improved latrines in the fragile contexts is still below the basic standard as defined by SDG 6. Female-headed households conduct household water treatment better than male-headed households. The majority of households in fragile contexts have no access to handwashing facilities due to poor infrastructure and limited water supplies.

It is recommended to build improved latrines to cover the existing backlog and ensure that every household has a toilet. Each village to have one borehole, which is connected to a pipe system. The pipe system will provide potable water close to the household, school, health and religious premises to ensure that people access potable water less than 500 meters away. Each household should own a latrine, given that 44.3% are not willing to share. In addition, the household latrines should have complete superstructures and lockable doors so that women can safely use them during day and night. Innovations such as haffir dams can be undertaken to allow harvested water to be drinkable. A haffir is an open pond fitted with plastic lining and linked to a filter well where people can access safe water to drink. As the water passes through the filter well-comprising sand, charcoal and gravel membrane, there will be a significant reduction of physical, chemical and biological contamination at the clean water well. The limitation is that the paper only focused on access to WASH facilities in fragile contexts. A cross-sectional analysis of biological, physical and chemical properties of water at the source and point of use is recommended for further research.

## Supporting information

S1 Questionaire (PDF)

S2 Questionaire (XLSX)

S3 Questionaire (XLSX)
