# Supplementary material for: Universal WASH coverage; what it takes for fragile states. Case of Jariban district in Somalia
Source: PLoS One. 2021 Feb 25;16(2):e0247417. doi: 10.1371/journal.pone.0247417 (PMC7906306; doi:10.1371/journal.pone.0247417)
Supplement: S1 Questionaire — (PDF) [file pone.0247417.s001.pdf]

Household questionnaire

Introduction

Hello. My name is ____. I am here on behalf of University of Venda to learn from you about Water, Sanitation and Hygiene (WASH). I am here to *ask you some questions with the aim of using the information to improve the quality of WASH services within the district. Information from this interview will be used for analysis but your identity will remain completely confidential. We request that you honestly answer all the questions but if at any point you decide not to respond, we shall respect your decision.*

Do we have your permission to continue?

QUESTIONNAIRE DETAILS

**100**	**Household Information**						
**101**	*A household is defined as a group of people who routinely eat out of same pot and live on the same compound (or physical location). It is possible that they may live in different structures*<br>**Please tell me of the people who normally live in your household**						
SN	Respondent code	Gender<br>1. Male<br>2. Female	Village name				
1							
2							
3							
4							
5							
6							
7							
8							
9							
10							

**WT**	**Water and Sanitation (WASH)**	
WT801	What is the source of water for members of your household? *Please circle the response*<br>1. Borehole<br>2. Public taps/stand posts<br>3. Protected dug well<br>4. Protected spring<br>5. Rain water<br>6. Water pond/dam<br>7. Water Berkards<br>8. Unprotected dug well<br>9. Others, specify	
WT802	How far (in kilometers) is the main source of water your household uses?<br>1. Less than 500 meters<br>2. 500meters-1 km<br>3. 1-2 kms<br>4. More than 2 kms	
WT803	How long (in minutes) does it take to fetch water and return home?<br>1. 0-30 minutes from the house<br>2. 30-60 minutes from the house<br>3. More than 60 minutes from the house<br>4. Don't Know<br>5. Others, specify	

WT804	When you reach the water source, how much time (minutes) do you have to wait for you to get water? (Queuing time).<br>1. 0-30 minutes<br>2. 30-60 minutes<br>3. More than 60 minutes<br>4. Don't Know<br>5. Others, specify	
WT805	On average, how many litres of water does your household use per day (for human use only)?<br>….. litres	
WT806	In the last one year, has water always been available from the source for your household?<br>1. Yes<br>2. No	
WT807	If no, which months are those when water was not available?<br>1. January<br>2. February<br>3. March<br>4. April<br>5. May<br>6. June<br>7. July<br>8. August<br>9. September<br>10. October<br>11. November<br>12. December	
WT808	How frequently has water not been available at the source during the past one year?<br>1. Once a week<br>2. Once a fortnight<br>3. Once a quarter<br>4. Once in six months<br>5. Once a year<br>6. None	
	**Water Treatment**	
WT809	Do you treat your water in any way to make it safer to drink?<br>1. Yes<br>2. No	
WT810	If yes, what do you usually use to treat the water?<br>1. Boiling<br>2. Use water treatment tablets(P&G tablets, water guard, chlorine, aqua tab)<br>3. Filtering with cloth<br>4. Solar disinfection<br>5. Others, specify	
WT811	If yes, how often do you treat your drinking water?<br>1. Rarely<br>2. Sometimes<br>3. Always	
WT812	If no, why?<br>1. Materials difficult to access<br>2. Boiling is time wasting<br>3. Water looks clean<br>4. Treating materials are expensive<br>5. Other specify	
WT813	If treatment materials are bought, how much do you pay for the treatment materials? (USD)	

WT814	How important to you is treating drinking water?<br>1. Not important<br>2. Important<br>3. Very important<br>4. Not sure/don't know	
WT815	When did you last treat your drinking water?<br>1. Today<br>2. Yesterday<br>3. Less than 1 week<br>4. One week ago or more/less than a month ago<br>5. One month ago or more<br>6. Don't remember	
**SH**	**Sanitation & Hygiene**	
SH824	Where do you dispose off human feacal matter? (Observe). *Please circle*<br>1. Pour flush latrine<br>2. Ventilated improved pit latrine<br>3. Pit latrine without slab<br>4. No facilities/bush<br>5. Others, specify	
SH825	Do you share this facility with other households?<br>1. Yes<br>2. No	
SH826	On which occasions do you use soap or ash when washing your hands? *Please do not prompt but circle all that apply.*<br>1. After use of the toilet/defaecation<br>2. After attending to child who has defecated<br>3. Before preparing food<br>4. Before eating<br>5. Before breastfeeding and feeding the child<br>6. After handling animals<br>7. Others, specify	
SH827	Please show me where members of your household wash their hands<br>1. **Observed:** Hand washing station with water + soap<br>2. **Observed:** Hand washing station with water + ash<br>3. **Observed:** Hand washing station with water only<br>4. **Observed:** Hand washing station with no water/no soap/no ash<br>5. No hand washing station in dwelling/yard/plot<br>6. Not observed: Permission withheld<br>7. Others, specify	
